# EEG-based workload estimation across affective contexts

**DOI:** 10.3389/fnins.2014.00114

**Published:** 2014-06-12

**Authors:** Christian Mühl, Camille Jeunet, Fabien Lotte

**Affiliations:** ^1^Institut National de Recherche en Informatique et en Automatique, Bordeaux Sud-OuestTalence, France; ^2^Laboratoire Handicap et Système Nerveux, University of BordeauxBordeaux, France; ^3^Laboratoire Bordelais de Recherche en Informatique (LaBRI)Talence, France

**Keywords:** workload, stress, brain–computer interface, classification, electroencephalography, passive brain computer interface

## Abstract

Workload estimation from electroencephalographic signals (EEG) offers a highly sensitive tool to adapt the human–computer interaction to the user state. To create systems that reliably work in the complexity of the real world, a robustness against contextual changes (e.g., mood), has to be achieved. To study the resilience of state-of-the-art EEG-based workload classification against stress we devise a novel experimental protocol, in which we manipulated the affective context (stressful/non-stressful) while the participant solved a task with two workload levels. We recorded self-ratings, behavior, and physiology from 24 participants to validate the protocol. We test the capability of different, subject-specific workload classifiers using either frequency-domain, time-domain, or both feature varieties to generalize across contexts. We show that the classifiers are able to transfer between affective contexts, though performance suffers independent of the used feature domain. However, cross-context training is a simple and powerful remedy allowing the extraction of features in all studied feature varieties that are more resilient to task-unrelated variations in signal characteristics. Especially for frequency-domain features, across-context training is leading to a performance comparable to within-context training and testing. We discuss the significance of the result for neurophysiology-based workload detection in particular and for the construction of reliable passive brain–computer interfaces in general.

## Introduction

The increasing complexity and autonomy of information systems rapidly approaches the limits of human capability. To avoid overload of the users in highly demanding situations, a dynamic and automatic adaptation of the system to the user state is necessary. Reliable knowledge about the user state, especially his workload, is a key requirement for a timely and adequate system adaptation (Erp et al., 2010). Examples are systems supporting air traffic control, pilots, as well as medical and emergency applications.

Conventional means of workload assessment, such as self-assessment and behavior, are intrusive or limited in their sensitivity, respectively (Erp et al., 2010). Physiological sensors, assessing for example the galvanic skin response (GSR) or elecrocardiographic activity (ECG), offer an unobtrusive and continuous measure that has been found sensitive to workload (Verwey and Veltman, 1984; Boucsein, 1992). In the last two decades, neurophysiological activity became popular as a modality for the measurement of mental states in general and of workload in specific. So-called “passive brain-computer interfaces” (pBCI, Zander and Kothe, 2011) are able to measure neuronal activity in terms of the electrophysiological activity of neuron populations as in the case of EEG or the oxygination of the cerebral blood flow as for functional near-infrared spectroscopy (fNIRS). Both approaches have been found informative regarding the detection of cognitive load (Brouwer et al., 2012; Solovey et al., 2012), and there is evidence for a partially superior sensitivity of neural measurements compared to other physiological sensors (Mathan et al., 2007) or self-report (Peck et al., 2013).

Most experiments on passive BCI use a very controlled approach, which naturally limits the range of real-world conditions they reflect. While this control is necessary to ensure the psychophysiological validity of the mental state detection, their results lack a certain ecological validity, they can not be generalized to other contexts. This might be one of the most impeding problems for the creations of passive BCI systems that work in the real world, since daily life is characterized by the variability of the conditions we function under. A prominent example are changes of affect while working, for example working under the pressure of an impending evaluation vs. work without pressure. A system that is supposed to work in such contexts needs to be calibrated and tested in them. Previous research in the domain of pBCI largely ignored the problem. To shed light on the interaction of mental state classification and change of affective context, we devised a protocol that recreates conditions of work, requiring different effort, during relaxed conditions and under psychosocial stress in a controlled environment. To study the resilience of a state-of-the art workload detection system to changes in affective context, we train subject-specific classifiers in either stressed or non-stressed context and test their performance within the same and in the other context.

In summary, the contributions of this paper for the study of the effect of affective context on workload classification are:

1. The creation and validation of a novel protocol to test interactions of workload classifier performance and affect^1^.
2. The design and evaluation of a workload classifier generalizing across affective contexts.
3. Quantifying the impact across affective context generalization on classification performance, with and without across context-training.

Below, we will give the reader some background to neurophysiology-based detection of workload under varying (affective) user states and its potential interactions with stress responses. Then, we will introduce the employed approaches to manipulate the user's mental state, the used devices, and the applied signal processing and classification algorithms. We will then report the nature of the found effects, discuss their relevance, and conclude with the general consequences and limitations of the presented findings.

## Related work

### Detection of workload from neurophysiology

Mental workload can be defined as (perceived) relationship between the amount of mental processing capability and the amount required by the task (Hart and Staveland, 1988). The closer the requirements are to the actual capabilities, the higher is the (perceived) workload. Therefore, a general strategy for workload manipulation is the manipulation of task demand or difficulty (Gevins et al., 1998; Grimes et al., 2008; Brouwer et al., 2012), though alternative strategies, such as the manipulation of feedback or participant motivation (Fairclough and Roberts, 2011), exist.

Already in 1998, Gevins et al. (1998) showed that EEG is a viable source of information regarding the workload of a person, enabling 95% accuracy when using about 30 s of signal. However, there are many factors that can affect the performance of classification algorithms, such as the number of training data available, their distribution, their separability between classes, the data signal-to-noise ratio, the similarity (in terms of data distribution) of the training data and testing data, etc. (Duda et al., 2001). The estimation of these performances also depends on the number of testing data available (Müller-Putz et al., 2008), and the way they are estimated (cross-validation, independent test set). Finally, more BCI-specific factors affect the performances, such as whether the classification is subject-specific or subject-independent (see, e.g., Lotte et al., 2009), which subjects are used (there is a huge between-subject variability), whether the training and testing data are from the same session (e.g., same day) or not, etc. (Lotte et al., 2007). In this regard, Grimes et al. (2008) showed that a number of factors, such as the numbers of channels, amount of training data, or length of trials, have a strong influence on classification performance of workload classifiers. For example, reducing the length of the signal from 30 to 2 s reduces the classification performance on two workload levels from almost 92% to about 75%. Similar tradeoffs between optimal and practical signal processing settings are reported for channel number and training time. Another work, by Brouwer et al. (2012), studied in a similar setup the feasibility of different types of features (i.e., from the time- and frequency-domain, and combined) to differentiate workload levels, finding that the different feature types work comparably well with accuracies of about 85% after 30 s. Reducing the signal length to 2 s reduced the accuracy to about 65%. Zarjam et al. (2013) showed that workload manipulated by an arithmetic task can be classified with a performance of 83% for seven workload levels. Walter et al. (2013) tested the generalization of workload classifier from simple tasks, such as go/no-go, reading span, n-back tasks, to complex tasks involving diagram and algebra problems. While they were able to train well-performing classifiers for the simple tasks, reaching performances of about 96% for two classes on signals of a few seconds length, a cross-testing of a workload classifier trained on a simple task to a complex task did not succeed. However, since in both studies the order of workload levels was not randomized, a temporal trend present in the features could have biased the results toward a higher accuracy. Overall, these studies show that the workload level can be classified from neurophysiological activity. Indeed, it has also been suggested that neurophysiological information is more sensitive than information from other physiological signals (Mathan et al., 2007). Most importantly, these studies show that different factors, mainly methodological differences in workload induction, signal acquisition and processing, can have significant influences on the classification results.

However, to date there have only been few studies regarding the influence of the mental state changes during training and testing on the classifier performance. For active BCI, Reuderink and colleagues studied the influence of frustration on left and right hand movement classification during a computer game, using freezing screens and button malfunctions as induction tools (see Reuderink et al., 2013). The resulting loss of control (LOC) during “frustrating” episodes, surprisingly led to higher classification performance than during normal, relaxed game play (Reuderink et al., 2011). Zander and Jatzev (2012) induced LOC in a similar way during a simple behavioral task, the RLR paradigm, which resulted in lower classification performance. For passive BCI and specifically for workload level detection, only Roy et al. (2012) tested the impact of fatigue on EEG signal characteristics and workload classification performance. With increasing fatigue, the differentiating signal characteristics diminished and, consequently, the classification performance declined. This lack of research on interactions between passive BCI and changes in user state is problematic, since BCIs in general have been found susceptible to changes in task-unrelated mental states during classification, such as attention, fatigue or mood. Specifically, it is believed that variations in task-unrelated mental states are partially responsible for what is called non-stationarities of the signal, the change of its statistical properties over time, and thereby the source of one of the most notorious problems for BCI (Krusienski et al., 2011; van Erp et al., 2012).

In the next section, we will briefly introduce the concept of stress, which is another possible contextual factor influencing workload estimation that is occurring during daily life and work, and thus might be a relevant source of variance for workload detection devices.

### Stress responses and workload

The psychophysiological concept of “stress,” was introduced in 1936 by Selye (1936) to describe “the non-specific response of the body to any demand for change.” In that sense, it is an organism's response to an environmental situation or stimulus perceived negatively—called a “stressor”—which can be real or imagined, that taxes the capacities of the subject, and thus has an impact on the body's homeostasis (that is to say that the constants of the internal environment are modified). To face the demand (i.e., to restore homeostasis), two brain circuitries can be activated during a “stress response cascade” (Sinha et al., 2003; Dickerson and Kemeny, 2004; Taniguchi et al., 2009): the sympatho-adrenomedullary axis (SAMa, also called the noradenergic circuitry) and the hypothalamus-pituitary gland-adrenal cortex axis (HPAa). On the one hand, the SAMa induces the release of noradrenaline which allows immediate physical reactions (such as increased heart rate and skin conductance, or auditory and visual exclusion phenomena) associated with a preparation for violent muscular action (Dickerson and Kemeny, 2004). On the other hand, the HPAa activation (which is lower) results in the releasing of cortisol the purpose of which is to redistribute energy in order to face the threat. Thus, more energy is allocated to the organs that need it most (brain and heart), while non-necessary organs for immediate survival (reproductive, immune and digestive systems) are inhibited. This stress response cascade ends when homeostasis is restored.

However, stress can be of different types, such as physical, psychological or psychosocial (Dickerson and Kemeny, 2004), each kind of stress being associated with a specific response. Indeed, physical stress, induced by extreme temperatures or physical pain for example, is associated with an increase of heart rate (Loggia et al., 2011), skin conductance (Boucsein, 1992; Buchanan et al., 2006) and subjective stress ratings but with only a weak cortisol response (Dickerson and Kemeny, 2004). These results suggest that this kind of stress induces an activation of the SAMa but only a weak activation of the HPAa. Psychological or mental stress, associated with difficult cognitive tasks, uncontrollability or negative emotions is associated with a weak release of cortisol (weak HPAa activation), but strong effects on heart rate and skin conductance (strong SAMa activation) (Boucsein, 1992; Reinhardt et al., 2012). Finally, psychosocial stress, triggered by a social evaluation threat (that is to say a situation in which the person's own estimated social value is likely to be degraded), and added to by a feeling of uncontrollability (in particular during the Trier Social Stress Task (TSST) Kirschbaum et al., 1993), has been shown to induce a strong activation of both the SAMa (Hellhammer and Schubert, 2012) and the HPAa (Dickerson and Kemeny, 2004).

Psychosocial stress and workload potentially can interact on physiological, neurophysiological and behavioral levels. Since workload can also be understood as the response to a particular type of psychological stressor, such as increased task demand, both concepts are associated with the activation of the sympathetic nervous system (see SAMa above). Furthermore, psychosocial stress and workload share also neurophysiological responses. From research in the neurosciences, and consistent with the notion of neural response systems, we know that stress has strong correlates in the EEG as well. One of the most prominent correlates of anxiety, as induced by psychosocial stress, is found in the alpha band, and specifically in brain asymmetry. Tops et al. (2006) proposed that cortisol administration (which simulates a stress situation) leads to a global decrease of cortical activity (except for the left frontal cortex in which activity is increased). However, other studies (Lewis et al., 2007; Hewig et al., 2008) showed that stress was associated with a higher activity in the right hemisphere, and that the right hemisphere activation was correlated with negative affect. For Crost et al. (2008), the explanation of these conflicting results would be that an association between EEG-asymmetry and personality characteristics, such as anxiousness, may only be observed in relevant situations to the personality dimensions of interest. For workload, on the other hand, we know that the alpha band plays a role in terms of increased sensory processing leading to decreased occipito-parietal alpha power (Gevins et al., 1998; Brouwer et al., 2012), as well as for frontal alpha asymmetry covarying with changes in engagement (Fairclough and Roberts, 2011). From a theoretical point of view, Eysenck and Derakshan (2011) suggested that increasing anxiety, for example due to psychosocial stress, has effects on different cognitive processes, leading to impaired processing efficiency and performance effectiveness. Specifically for workload-related processes, their “attentional control theory” suggests that anxiety impairs efficient function of inhibition and shifting mechanisms of the central executive, subsequently decreasing attentional control and increasing distraction effects of irrelevant stimuli. However, these deficits might not necessarily lead to decreases of performance if they are compensated by alternative strategies, such as enhanced effort.

Summarizing, increases in workload, as induced by higher task demand, can be subsumed under the concept of psychological stress and have been found to lead to increasing physiological and neurophysiological activity that has also been found responsive to anxiety as induced by psychosocial stress. Furthermore, cognitive theories propose links between anxiety and pre-attentional and attentional cognitive processes, which are expressed in behavior and physiology. Due to these possible interactions of workload and stress, it seems relevant to experimentally study the effect of stress on workload detection.

## Research questions

The work on the effects of potential contextual factors, such as moods or fatigue, on the stability of BCI performance, and the physiological and psychological links between stress and cognitive processes suggests that stress can be a relevant factor influencing the classification of workload levels. In more general, the findings of context-dependency of BCI performance make it seem imperative to explore the effect of factors, such as mood, on brain signals and classifier performance, to gain insight into the relevance of task-unrelated mental states on classifier performance, and to find ways to render classifiers robust against such changes. Specifically, for the development of reliable passive BCIs in the wild, those functioning robustly in private or work environments, the influence of contextual changes of mental states that are predominant in the context of application have to be explored. That is why we test the robustness of three workload classifiers, using features from either frequency-, time-, or both domains, to the influence of (psychosocial) stress. We let participants work under different levels of workload, while either under the impression of being observed and validated, or while being relaxed and free from this kind of pressure. We are interested in the effect of the contextual manipulation of stress on the classifier performance and in testing cross-context training as a simple and straightforward remedy to the problem. Thus, we address the following questions:

*Q1: Can we induce stress and workload in a controlled manner?* We validated stress and workload manipulation of our experimental protocol using participants' self-assessments, behavioral performance, and physiological indicators of sympathetic nervous system (SNS) activation (i.e., GSR, ECG). Stress is expected to increase perceived anxiety and SNS activity, while workload increase should be reflected in increased perceived arousal and mental effort, decreased performance, as well as increased SNS activity (Verwey and Veltman, 1984; Boucsein, 1992).

*Q2: Can we train a workload classifier based on the data collected via this protocol?* To ensure that we are using a state-of-the-art workload classifier, we trained the classifier on all data, irrespective of context, as done in conventional studies. We expect a performance of about 70% as shown by Grimes et al. (2008) and Brouwer et al. (2012) under similar conditions.

*Q3: Does the classifier generalize across affective contexts, and if so, how well?* To study the effect of different affective contexts on the classification performance, we compared the results from classifiers trained in either stressful or non-stressful context and applied it then to test data from the same (“within”) or the other context (“across”). We expect a higher “within” compared to “across” performance to indicate the difficulty of the classifier to generalize.

*Q4: Does training based on multiple context render the classifier resistant against changes in affective context, and if so, how resistant?* To test if the training with combined data from both affective contexts is effecting the classifier's capability to generalize, we compare the performance depending on the training context (“single,” that is training on only stress or non-stress context, or “combined,” that is training over contexts) and expect higher performance for a classifier trained on data from the combined contexts.

## Materials and methods

As mentioned before, we designed a protocol in which subjects had to do cognitive tasks involving two levels of mental workload, manipulated via task difficulty, while being exposed to two levels of psychosocial stress. We used the EEG signals collected with this protocol to design and assess a workload classifier across different stress conditions. This section describes in details the subjects involved, the protocol and the method to validate it, the EEG-based workload classifier used and the evaluations performed with it.

### Participants

Twelve female and twelve male participants were recruited for our experiment. The participants were between 18 and 54 years old, with a mean age of 24.7 ± 7.9, and except four all were right-handed. Educations varied between high school degree and Ph.D., with a mean education of 3.1 ± 2.4 years after high school. To be admitted, people had to be at least 18 years, to speak the local language and to sign an informed consent. Furthermore, non-inclusion criteria were applied: bad vision, heart condition, neurological or psychological diseases, and affective troubles. Moreover, people were asked to select a time for the experiment in which they would feel alert. Finally, we asked them not to drink coffee and tea less than 2 h before the experiment.

### Material

**Figure 1:**
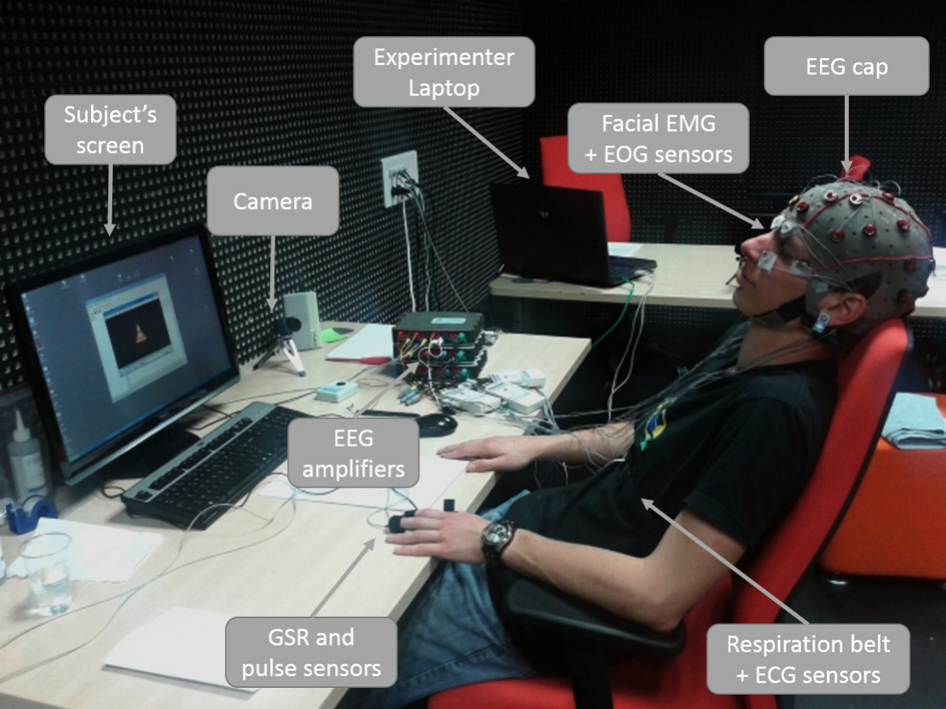
**A fully wired participant in the experimental environment during the relaxation induction period**.

For our recordings, we used the following sensors: ElectroEncephaloGram (EEG, 28 active electrodes in a 10/20 system without T7, T8, Fp1, and Fp2), ElectroCardioGram (ECG, two active electrodes), facial ElectroMyoGram (EMG, two active electrodes), ElectroOculoGram (EOG, four active electrodes), breath belt (SleepSense), pulse (g.PULSEsensor), and a galvanic skin response sensor (g.GSRsensor). All sensors were connected and amplified with three synchronized g.USBAmp amplifiers (g.tec, Austria). The workload task was designed in the Presentation software (Neurobehavioral Systems, www.neurobs.com/presentation) and EEG signals were recorded and visually inspected with Open ViBE (Renard et al., 2010). Figure 1 shows a participant sitting fully-wired in the experimental environment.

**Figure 2:**
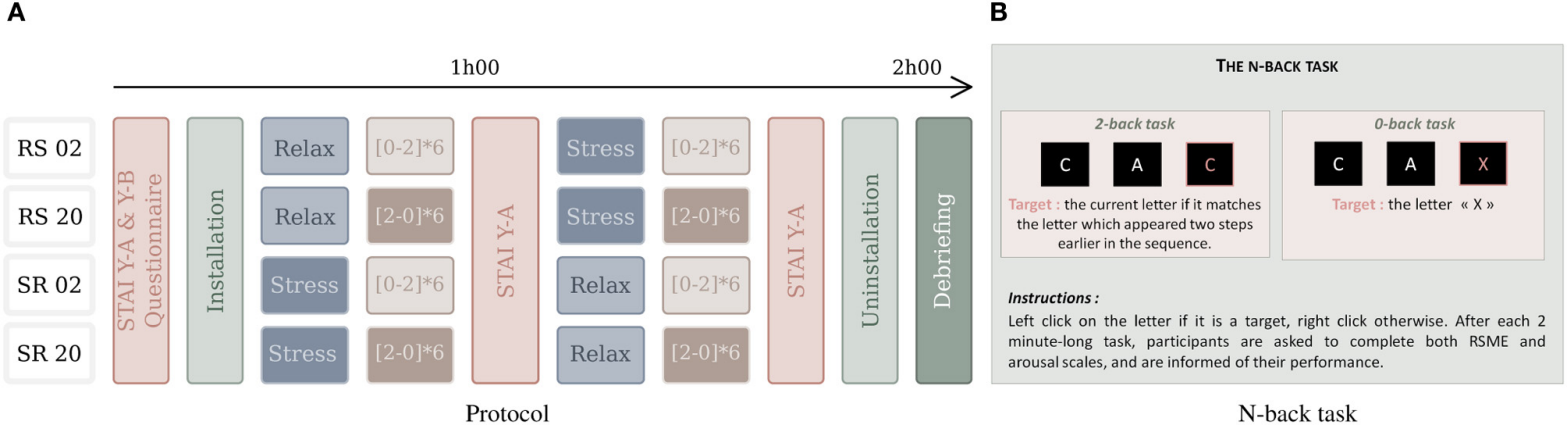
**The experimental protocol **(A)** with four scenarios, used for counterbalancing the order of conditions: the easy (0-back task) and difficult (2-back) workload tasks that follow the (R)elax and (S)tress induction procedures**. “RS02” means that relaxation context is followed by stress context and that the interleaved workload blocks start with the 0-back condition. The N-back task **(B)** requires responses to target and non-target characters.

Subjects were first asked to sign an informed consent and to fill out three questionnaires: one assessing personal characteristics (such as gender, age and education) and form Y-A (anxiety state) and Y-B (anxiety trait) of the State-Trait Anxiety Inventory (STAI) (Spielberger et al., 1970) (see below for details). Then, all the sensors were installed and a 3 min baseline recorded. To avoid order effects, we counterbalanced the order of stress and relax condition (affective context) and 0-back and 2-back task (workload blocks), resulting in four scenarios (see Figure 2A). Each scenario was composed of 12 workload blocks in the stressful and 12 workload blocks in the relaxed context. The scenarios therefore begin with either relaxation or stress induction, and the workload blocks either start with the low workload (0-back) or high workload (2-back) condition. In each affective context, the subject performs, in alternating order, six times each workload condition (low/high) (6 × 2 × 2 = 24 min per block), with a short break after six tasks (i.e., after about 12 min). After each context was absolved, that is after the induction phase and the 12 workload blocks, the STAI form Y-A questionnaire was administered again to assess the anxiety state. Finally, the sensors were removed and the participant was debriefed about the aim of the experiment.

#### Stress and relaxation inductions

In order to manipulate stress, we used a stress-induction protocol based on the Trier Social Stress Task (TSST) (Kirschbaum et al., 1993) and a relaxation condition using a resting phase, music and/or videos. The stress-induction protocol is composed of three parts lasting together about 15 min and it requires the participation of three people, “the committee,” who are presented as being body language experts. In the first part, a member of the committee asks the subject to prepare, during 5 min, a fake job interview for a position fitting the professional profile of the subject. During the second part, the committee asks the person to do this job interview and to speak about himself for 5 min. They tell the subject that he is filmed for a future behavioral analysis and take notes during the whole interview. The committee acts as being serious and neutral/unresponsive toward the subject. The third part is a 3 min long arithmetic task (the subject has to count from 2083 to 0 by steps of 13) and to begin again at any mistake or hesitation. At the end of this protocol, in order to keep the stress level high, the committee tells the subject he will be filmed during the workload tasks and that he will have to do another interview, which will be longer, and a self-evaluation based on the recorded film material after it. Furthermore, during the experiment, participants are receiving visual feedback about their performance in the workload tasks. During the stress condition, these feedbacks have been modified to display a performance 5–10% below their actual performance. Thereby, this protocol includes psychosocial stress and uncontrollability in order to maximize the chance to trigger a stress response for all the participants (Dickerson and Kemeny, 2004). On the other hand, the goal of the relaxation induction was to create a condition (referred to as “relax” condition) in which participants would be able to relax and thus execute the workload task without the influence of additional psychosocial and psychological stressors. To allow for an effective relaxation, participants were allowed to choose between resting in silence or select music/videos that would help them to feel calm (Krout, 2007). In order to measure the level of anxiety of the subjects and thereby to validate the stress/relax manipulation, the “State Trait Anxiety Inventory” (Spielberger et al., 1970) is used. It is composed of two scales of 20 propositions each: STAI form Y-A and STAI form Y-B. STAI form Y-A score measures anxiety state and is increased when the person currently experiences psychological stress. A college student (female/male) has a mean state anxiety index of 35/36, while values higher than 39/40 have been suggested to detect clinically significant symptoms (see Julian, 2011).

#### Workload tasks

We used the n-back task (Kirchner, 1958) as workload task (see Figure 2B), as it is easy to modify workload while keeping visual stimulation and behavioral motor requirements the same. Similar to Grimes et al. (2008) and Brouwer et al. (2012), we decided for a manipulation of task-difficulty to manipulate workload. Specifically, we used 0-back (low workload) and 2-back (high workload) varieties of the n-back task, which were presented in blocks of 2 min each. In both tasks, a stream of 60 white letters appears on a black background on the screen. Each letter is presented for 500 ms, followed by an inter-stimulus interval of 1500 ms. Among these letters, 25% are targets. In both tasks, when a letter appears, the subject is asked to perform a left mouse click if this is a target letter, and a right mouse click otherwise. For the 0-back task, the low workload condition, the target is the letter “X”: each time an “X” appears, the subject has to do a left click, and in all the other cases he has to do a right click. For the 2-back task, the high workload condition, the subject has to do a left click if the letter that appears is the same as the one preceding the last letter. For example, if the sequence “C A C” appeared, the second “C” would be a target. At the end of each 2-min block, the subject has to report his level of arousal (on a scale from 1 to 9) (Bradley and Lang, 1994) and the perceived effort necessary to perform the task (Rating Scale of Mental Effort—RSME, Zijlstra, 1993). Finally, a screen with his performance during the block (see section 4.3.2) appears. As mentioned before, during the stressful condition, this displayed performance is lower than the actual performance to induce additional uncertainty.

### Protocol validation methods

#### Self-assessment data

To investigate the effect of the psychosocial stress induction on the STAI score, we computed an ANOVA with this score in the three factor-levels “baseline,” “after relaxation,” and “after stress induction.” To assess the effect of both stress and workload manipulation, we conducted 2 (stress) × 2 (workload) ANOVAs for the averaged-over-blocks ratings on the arousal scale of the SAM and on the RMSE.

#### Behavioral data

To investigate the effects of the experimental manipulations on behavior, we calculated the performance per block based on the number of true positive (*TP*), true negative (*TN*), false negative (*FN*), and false positive (*FP*) responses resulting from the button presses within the n-back task (left click for targets, right click for non-targets) using the following equation: $Perf=\frac{\left(TP+TN\right)}{\left(TP+TN+FP+FN\right)}$. As for ratings, we analyzed the data in a 2 (stress) × 2 (workload) ANOVA.

#### Physiological data

Physiological responses were analyzed with respect to heart rate (HR) and galvanic skin response (GSR). Before applying statistical methods, the GSR data was pre-processed by extracting the mean GSR value (μS) for each block and then averaging these values over blocks as described above. The ECG signal was band-pass filtered between 5 and 200 Hz, applying a notch-filter 48–52 Hz to reduce power line noise, before mean HR for each of the blocks was extracted. As for the former analyses, we analyzed the data with a 2 (stress) × 2 (workload) ANOVA. We are reporting data as significant if *p* < 0.05 and as trend if *p* < 0.1. For all ANOVAs partial eta squared values (*η^2^_p_*) are calculated as a measure of effect size.

### EEG signal processing

**Figure 3:**
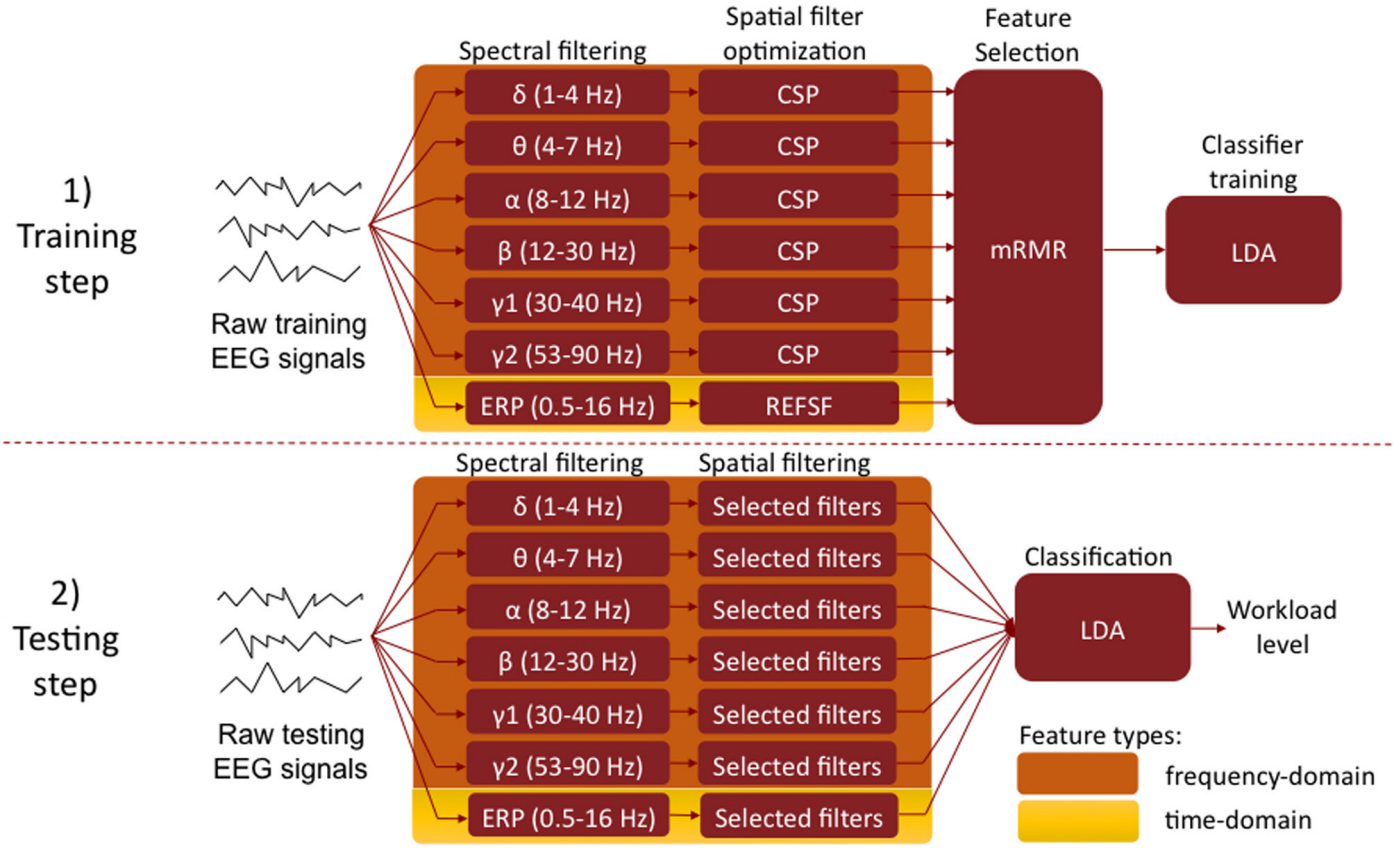
**Machine learning approach to workload level classification from EEG signals. Top:** training set, aiming at identifying the relevant frequency bands (i.e., spectral filters) and channels (i.e., spatial filters), using the Filter Bank CSP and REFSF approach. **Bottom:** testing set, using the optimized spectral and spatial filter to estimate the workload level from an unknown EEG trial. (CSP, Common Spatial Patterns; REFSF, regularized Fisher spatial filter; mRMR, maximum Relevance Minimum Redundancy; LDA, Linear Discriminant Analysis).

Our system aims at estimating the level of mental workload of the user from its EEG signals. To do so, we employed a machine learning approach based on state-of-the-art algorithms developed for Brain-Computer Interfaces (BCI) technologies (Lotte et al., 2007; Blankertz et al., 2008; Ang et al., 2012). This section describes the way EEG signals were preprocessed and segmented into trials, the machine learning algorithms used as well as the approach followed for the evaluating our method (see Figure 3 for a schematic overview of these procedures).

#### EEG preprocessing and segmentation

We first cleaned signals from eye movements (EOG) contamination using the automatic method proposed in Schlögl et al. (2007). The EEG signals from each 2 min n-back task were then divided into 60 EEG trials, i.e., one EEG trial per letter appearance. More precisely, each EEG trial was defined as starting at a letter appearance onset and ending 2 s later, i.e., just before the next letter appearance. This resulted in 60 EEG trials per task, i.e., 720 trials per workload level (360 trials in the stressful condition, 360 in the non-stressful condition). Among them, trials corresponding to target letters were discarded in order to avoid confounding and interfering effects that may result from Event Related Potentials (ERP—notably a P300) likely to be triggered by target identification. This left 540 trials per workload levels (270 trials per psychosocial stress condition).

#### Machine learning algorithms

In order to estimate workload levels from EEG signals, we investigated two different types of neurophysiological information: (1) oscillatory activity and (2) Event Related Potentials (ERP), both of which having been shown to be useful for such a task (Brouwer et al., 2012). We set up state-of-the-art signal processing pipelines in order to estimate workload using these two types of information, both individually and in combination (see Figure 3). They are described below:

***Oscillatory activity.*** To classify low mental workload vs. high mental workload in EEG signals based on oscillatory activity, we used a variant of the Filter Bank Common Spatial Patterns (FBCSP) algorithm (Ang et al., 2012) in order to learn optimal spatial and spectral features, i.e., EEG frequency bands and channels. The FBCSP is one of the most efficient algorithms to extract spatio-spectral features from EEG signals. It was indeed the algorithm used by the winners of the last BCI competition on all EEG data sets (Ang et al., 2012; Tangermann et al., 2012), showing the superiority of this method over other approaches. The FBCSP-based approach we employed works as follows. The first step—the training step—consists in identifying the most relevant frequency bands (i.e., spectral filters) and EEG channels (i.e., spatial filters), using examples of EEG signals from the high and low workload conditions (see below for details on the definition of the training sets). To do so, we first filter each training EEG trial into multiple frequency bands using a bank of band-pass filters. Here we used band-pass filters in the following frequency bands, which correspond to classical EEG rhythms: δ (1–4 Hz), θ (4–8 Hz), α (8–12 Hz), β (12–30 Hz), γ (30–47 Hz), and high γ (53–90 Hz). Then for each of these bands, the band-pass filtered EEG trials are used to optimize spatial filters, i.e., linear combinations of the original EEG channels. These spatial filters are optimized using the Common Spatial Pattern (CSP) algorithm (Blankertz et al., 2008), which finds the optimal channel combination such that the power of the resulting spatially filtered signals is maximally discriminant between the two conditions (here, low and high workload). We optimize 12 (6 pairs) such CSP filters for each frequency band. Then, the power of the spectrally and spatially filtered EEG signals is used as features, resulting in each EEG trial being described by 72 features (12 CSP filters × 6 frequency bands). From these 72 features, the 18 most relevant ones are selected using the maximum Relevance Minimum Redundancy (mRMR) feature selection algorithm (Peng et al., 2005). This amounts to selecting the 18 most relevant pairs of spectral and spatial filters. Finally, the 18 selected power features are used to train a shrinkage Linear Discriminant Analysis (LDA) classifier (Blankertz et al., 2010; Lotte and Guan, 2010) to discriminate low workload EEG trials from high workload ones. This concludes the training step. For testing, i.e., to predict the workload level of a given EEG trial, the EEG signals are first filtered using the 18 selected pairs of spectral and spatial filters, then the power of the resulting signals is computing and given as input to the previously trained LDA classifier whose output indicates the workload level (high or low).

***Event related potentials.*** To classifiy low mental workload vs. high mental workload in EEG signals based on ERP, we first band-pass filtered the signals between 0.5 and 16 Hz, and downsampled them to 36 Hz, to reduce the signal dimensionality. We only used the first second of EEG signals from each trial (i.e., the first second after letter presentation in the N-back task) to analyse ERP, i.e., 36 samples per channels. Then, based on these 1-second of EEG signals from the training set, we learned optimal spatial filters for the discrimination of ERP based on EEG samples, by using the Fisher Spatial Filters (FSF) proposed by Hoffmann et al. (2006). We extracted 6 such spatial filters, which resulted in 216 features (6 filters × 36 EEG samples per filter), using a regularization parameter λ = 0.4 for optimizing the FSF for all subjects. We finally selected 18 features (i.e., 18 EEG samples) out of these 216 initial ones, using mRMR feature selection. These 18 selected features were used to train a shrinkage LDA. For testing, the EEG signals were preprocessed in the same way (i.e., band-pass filtered in 0.5–16 Hz and downsampled to 36 Hz), spatially filtered using the 6 Fisher Spatial Filters optimized during training, and the 18 resulting selected features were used as input to the previously trained LDA classifier whose output indicates the workload level (high or low).

***Combination of oscillatory activity and ERP.*** In order to combine both oscillatory activity and ERP information, we extracted 18 FBCSP features as described above and 18 ERP features, as described above as well, from each trial. These 36 features were concatenated into a single feature vector, which was used as input to a shrinkage LDA classifier.

#### Evaluation scheme

The performance of our workload-level estimator was assessed using sixfold stratified Cross-Validation (CV), separately for each subject. This means the data from each subject was divided into six parts, each part containing the same number of trials from each class (high/low workload). Five of these parts were used for training, i.e., to identify the relevant spectral and spatial filters, as well as to train the LDA classifier. The 6th part was used for testing the resulting workload-level estimator for that subject. This process was repeated six times, with each part used exactly once as the testing set. For three subjects we used only three- and fourfold CV due to missing blocks in the end of the recording. The performance, here the classification accuracy (i.e., rate of trials with correctly estimated workload-level), hence obtained on each testing part are then averaged to give a final performance of the workload-level estimator for that subject.

The goal of our work is to design a generic workload-level estimator, usable in practice, i.e., that can work across different affective contexts (here, different psychosocial stress levels). To do so, we performed different evaluations to estimate (1) the general performance of our system, independently of the affective context; (2) how it behaves *within* a given affective context; (3) how it behaves *across* different affective contexts, i.e., can a workload-level estimator calibrated on data from a given affective context (e.g., a relaxed condition) be used to estimate workload in another affective context (e.g., a stressful condition), (4) if effects of time can explain across-context classification performance loss, and (5) whether calibrating our system with data from different affective contexts makes the system better or worse, even if used in a single affective context. Different sub-parts of the data were thus used for training and testing within our CV scheme, in particular:

1. **General performance estimation:** This is the overall evaluation, in which we used all the data, from both affective contexts, i.e., with EEG trials from both the relaxed and the stressful conditions. Therefore, within each fold of the CV, 20 blocks (i.e., 900 trials) were available for training, and 4 blocks (i.e., 180 trials) were available for testing. The number of trials from each workload-level (high/low) and each psychosocial stress (relaxed/stressful) was balanced in both the training and testing set.
2. **Within affective context performance estimation:** This evaluation assessed the performance of our system when calibrated on a single affective context and tested on the same affective context. This is the evaluation generally performed in previous works, in which a single affective context is considered. Therefore, in each fold of the cross-validation, 10 blocks (i.e., 450 trials) were available for training, all coming from the relaxed (resp. stressful) condition, and 2 blocks (i.e., 90 trials) were available for testing, all coming as well from the relaxed (resp. stressful) condition. The number of trials from each workload-level was balanced in both the training and testing set.
3. **Across affective context performance estimation:** This evaluation assessed the performance of our system when calibrated on a given affective context and tested on a different affective context. This evaluation is usually ignored in current workload-level estimation works. Previous works indeed implicitly considered that the user was always in the same affective state, which is very unlikely in practice and can thus compromise the usability of the system. Therefore, in each fold of the cross-validation, 10 blocks (i.e., 450 trials) were available for training, all coming from the relaxed (resp. stressful) condition, and 2 blocks (i.e., 90 trials) were available for testing, all coming from the other affective context i.e., the stressful (resp. relaxed) condition. The number of trials from each workload-level was balanced in both the training and testing set.
4. **Investigation of time effects on classifier performance:** To rule out that a difference between within-context and across-context training is merely caused by the time passing between affective contexts, we devised an analysis similar to the above two analyses, but with first and second half of each context instead of relax and stress context. Therefore, we trained our classifiers on the data of 4 blocks and tested them on 2 blocks from either the same or the other half of the context. This was done in a threefold cross-validation scheme and resulted in two within and two across classification performance values (one from 1st half to second half, and one backwards) for each affective context. These were averaged over the affective contexts and yielded one value for the workload classification accuracy for within- and across-context (i.e., “half”) per participant per half^2^. For a genuine effect of affective context instead of an effect of simply the time passing between both contexts, the “within vs. across halfs” performance loss for a classifier that was only trained on one half should be smaller compared to the loss between “within vs. across affective context” performance loss for a classifier that was only trained on one affective context.
5. **Calibration across affective context performance estimation:** When considering different affective contexts, an interesting question is whether using data from different contexts to calibrate the workload-level estimator will make it better or worse, notably as compared to the within affective context evaluation. Indeed, on the one hand, using data from different contexts can force the machine learning approach to identify workload indices that are invariant to the affective context, thus improving the system, but on the other hand it adds more noise and variability to the data, which can impede the machine learning process. Therefore, with this evaluation, in each fold of the cross-validation, 20 blocks were available for training, coming from both the relaxed and stressful condition, and 2 blocks were available for testing, coming from either the stressful or the relaxed condition (but not both). To ensure that the comparison of this approach with the within-context approach is fair, we had to use the same number of training trials for each approach. Indeed, using all the trials available in the 20 training blocks would mean using more training trials than in the within-context evaluation, which could result in higher performance simply due to a larger number of training trials. Therefore, for this last evaluation, we randomly selected 6 blocks from each context for training, from 4 of which all trials were used, while we selected every other trial from the remaining 2 blocks to keep the workload classes balanced within context. Further two blocks were selected from each context for testing. This procedure was repeated six times for a cross-validation comparable to the within-/across context evaluation.

## Results

In this section, we first present the validation analysis, suggesting that our protocol indeed induced different levels of workload and stress (Q1). Then the results of the EEG-based workload classification over, within, and across affective contexts are presented, showing that a state-of-the-art subject-specific workload classifier (Q2) has difficulty generalizing over affective contexts (Q3), but can be rendered less context-sensitive by calibration across affective contexts (Q4).

### Validation of the protocol

#### Subjective indicators

**Figure 4:**
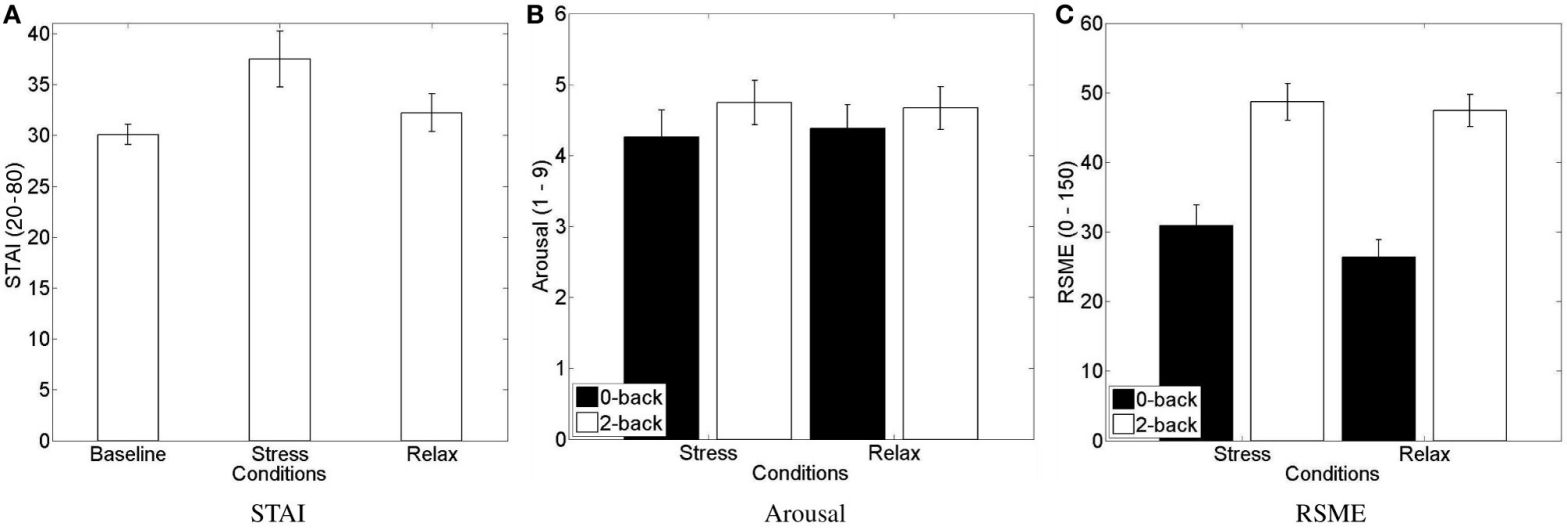
**Mean and standard error of mean of subjective stress level assessments. (A)** STAI form Y-A scores, **(B)** SAM arousal scale, and **(C)** RSME. **(A)** Shows significant increase of perceived stress during the stressful condition compared to the baseline and the relax condition. **(B,C)** Show an increase of perceived arousal and mental effort for the 2-back compared to 0-back task.

Each subject filled in three “STAI form Y-A” (state) questionnaires: one at the beginning (*STAI_BL_*) of the experiment and one in the end of each affective context, that is after performing the n-back tasks under stress or relax condition (stress: *STAI_S_*; relax: *STAI_R_*) (see Figure 4A). Three data sets were excluded due to incompleteness. A repeated-measures ANOVA (*N* = 21) with the factor levels “baseline,” “stress,” and “relax” showed a significant difference of perceived anxiety between the conditions [*F*_(2, 20)_ = 3.6225, *p* < 0.05, *η^2^_p_* = 0.108]. We conducted a *post hoc* analyses using paired *t*-tests with the hypothesis that subjectively perceived anxiety increases due to the stress induction procedure relative to baseline and relaxation condition. The results suggest that the stress-induction protocol indeed increases anxiety compared to baseline and relaxation condition, and keeps it significantly higher until measured in the end of the affective context (see Figure 4A): *STAI_S_* scores (mean = 37.5 ± 12.6) are significantly higher [*t*_(20)_ = 2.87, *p* = 0.01] than *STAI_BL_* scores (mean = 30.1 ± 4.6) and they are significantly higher [*t*_(20)_ = 2.37, *p* = 0.028] than *STAI_R_* scores (mean = 32.2 ± 8.6). This increased anxiety seems mainly due to the interview and the apprehension of a final evaluation, rather than due to the n-back task as such: we found no difference between *STAI_R_* and *STAI_BL_* [*t*_(20)_ = 1.27, *p* = 0.22], that is when they performed the n-back tasks knowing that there would be no evaluation.

We furthermore asked the subjects after each block to rate their arousal on the respective scale of the Self-Assessment Maneken (see Figure 4B) and to rate the mental effort on the Rating Scale Mental Effort (see Figure 4C). Two data sets were excluded due to incompleteness. We submitted the data of each scale to a 2 (stress) × 2 (workload) repeated-measures ANOVA. Regarding the subjectively perceived arousal, we only found a main effect of the workload manipulation [*F*_(1, 21)_ = 4.444, *p* = 0.047, *η^2^_p_* = 0.175] with higher perceived arousal for the 2-back task (mean = 4.7 ± 1.4) compared to the 0-back task (mean = 4.3 ± 1.7). Regarding the subjectively perceived workload, we only found a main effect of the workload manipulation [*F*_(1, 21)_ = 63.216, *p* < 0.0001, *η^2^_p_* = 0.751] with higher perceived effort for the 2-back task (mean = 48.1 ± 11.5) compared to the 0-back task (mean = 28.6 ± 12.9).

#### Objective indicators

**Figure 5:**
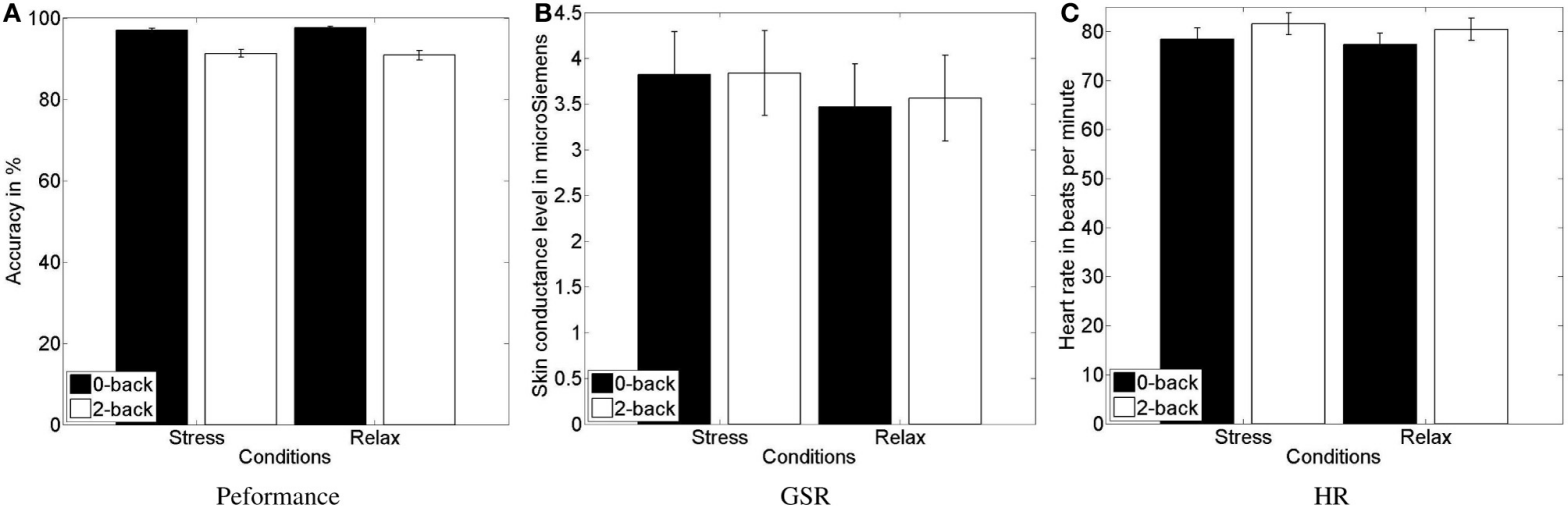
**Mean and standard error of the task performance (A), showing the decreased performance in high vs. low workload conditions, and of GSR response (B) and heart rate (C), indicating higher physiological activity in response to the psychosocial stress and workload manipulation, respectively**.

For the analysis of the objective indicator of behavioral performance, we logged all responses and computed the task accuracy for each task block (see Figure 5A). Two data sets were excluded due to incompleteness. We submitted the accuracy to a 2 (stress) × 2 (workload) repeated-measures ANOVA. As for the subjective indicators of perceived arousal and effort, we found a main effect of the workload manipulation [*F*_(1, 21)_ = 65.251, *p* < 0.0001, *η^2^_p_* = 0.757] with higher accuracy for the simple 0-back task (mean = 97.3 ± 2.0) compared to the hard 2-back task (mean = 91.1 ± 4.8).

As a further objective indicator, we computed skin conductance level and heart rate. Four data sets were excluded due to incompleteness. For heart rate analysis a further data set was excluded due to malfunctioning sensors. We submitted the data of the physiological signals to a 2 (stress) × 2 (workload) repeated-measures ANOVA. For GSR (see Figure 5B), we found an increase of the skin conductance level [*F*_(1, 19)_ = 4.4806, *p* = 0.048, *η^2^_p_* = 0.191], indicating higher sympathetic arousal during the stress condition (mean = 3.83 ± 2.05) compared to the relax condition (mean = 3.52 ± 2.07). Skin conductance level increased for high compared to low workload condition as well, however, not significantly. For HR (see Figure 5C), we found a trend toward an increase of the heart rate [*F*_(1, 18)_ = 3.2123, *p* = 0.089, *η^2^_p_* = 0.151], indicating higher sympathetic arousal during the stress condition (mean = 79.41 ± 10.23) compared to the relax condition (mean = 78.30 ± 10.08). More importantly, we found a highly significant effect of the workload manipulation on HR [*F*_(1, 18)_ = 36.1431, *p* < 0.0001, *η^2^_p_* = 0.667], with a higher HR for the more challenging 2-back task (mean = 80.4 ± 9.89) compared with the relatively easy 0-back task (mean = 77.27 ± 10.19).

In summary, we found evidence for the validity of the stress and workload induction (Q1) in both, the subjective (questionnaires) and objective (performance and physiological sensors) measures. This ensures that calibrating and evaluating a workload classifier on the EEG recorded with this protocol is meaningful.

### Classification of EEG

#### General performance estimation

In this section we report the general classification performance for a training on the whole data set, showing that our setup is state-of-the-art compared to similar studies hence positively answering question Q2. Specifically, we obtained performances similar to the best performances that were presented more recently with the n-back task paradigm and with 2 s short trials by Grimes et al. (2008) and Brouwer et al. (2012). The data of two participants was excluded due to incompleteness and of another one due to malfunctioning EEG sensors.

**Figure 6:**
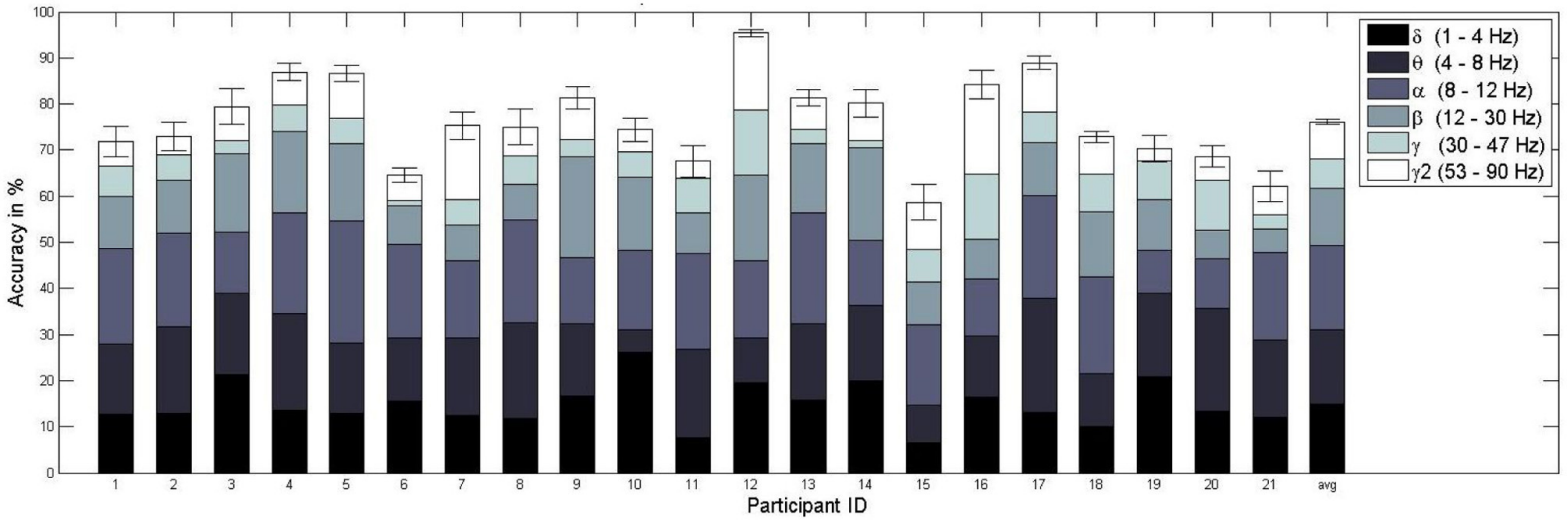
**Mean and standard error of the workload classification performance (sixfold cross-validation) per subject**. The different colored subdivisions within each bar represent the percentage (total bar height = 100%) of features selected from a specific frequency band (delta, theta, alpha, beta, gamma, gamma2). For example, for subject 1 on average 9% of the features were chosen from the delta range. The last bar represents the mean classification accuracy over subjects and the average contribution from the frequency bands over subjects.

For the training and testing on the basis of all available data, those trials recorded during stress *and* relax context, we achieved an average classification accuracy of 76.1% when using only frequency-domain features, with performances between 58.7% and 95.4% (see Figure 6). According to Müller-Putz et al. (2008), we determined the above chance-level performance via a binomial test. For a two-class problem and given the number of 1080 trials used in our sixfold cross-validation scheme, the chance-level is at 53.1% for *p* = 0.05. Consequently, the classification performance was above chance for each subject, with a highly significant better-than-random performance for the average result over all subjects (*p* « 0.0001).

Subsequently, we tested the previously observed increase of performance for increasing decision intervals, that is when more data is available for testing (Grimes et al., 2008; Brouwer et al., 2012). A majority vote over the classifier decisions for all 45 relevant trials of a given block, using only frequency-domain features, leads to an accuracy of 96%, well over the 71% chance-level resulting from a binomial test on the basis of 24 decisions (one per block). For time-domain features, we observed an average accuracy of 74% for 2 s trials (of which only the first was used), and 96% for the judgement after 45 trials. For both feature varieties in combination, the 2-second accuracy was the highest with 80.4%, though the block-wise accuracy was only 94.4%. Since all accuracies are well over chance level the used classification schemes enable for a solid classification performance for all feature varieties with the combined frequency- and time-domain features performing best for short estimation intervals and separate feature varieties performing best for the long decision intervals.

From a scientific point of view it is necessary to know about the source of the classification performance: is the information of neural origin or is it derived from muscular activity that is known to contaminate higher frequency bands of the EEG (Goncharova et al., 2003)? Although this question is often eluded in previous works (Grimes et al., 2008), we tried to answer it by first computing the percentage of the features selected from each frequency band in the FBCSP algorithm. As Figure 6 indicates, the majority (about 65%) of features selected with the mRMR feature selection algorithm employed came from lower frequency bands (i.e., delta, theta, alpha). However, the remaining 35% originated in high frequency bands, those over 12 Hz (beta, gamma, gamma2). To ensure that the classifier performance does rely on neuronal sources and not on muscle activity, we repeated the workload classifier evaluation excluding these potentially contaminated high frequency bands, both for training and testing. We achieved a somewhat lower, but again much better-than-random (*p* « 0.0001) classifier performance of 74.2%, with accuracies between 53.9% and 88.2%. This suggests that our workload classifier does rely mostly on neural information from low frequency bands.

#### Within- vs. across-context estimation

**Figure 7:**
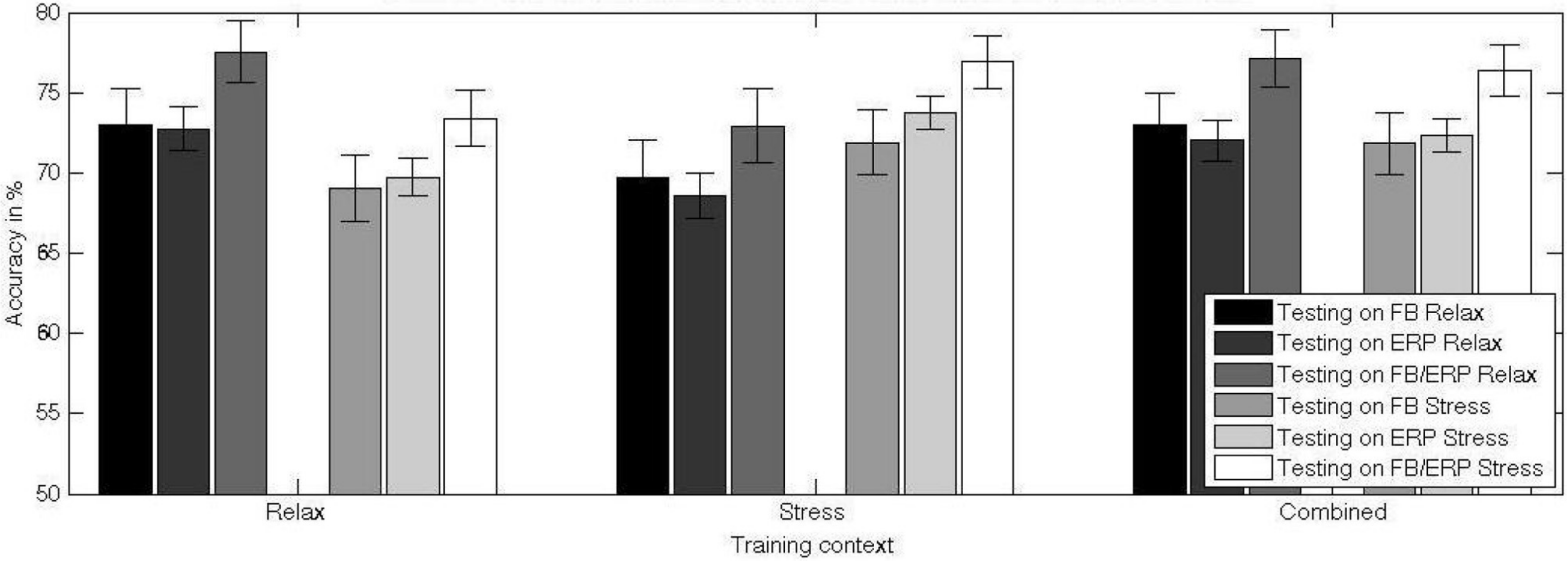
**Mean and standard error of the mean of the classification performance of a classifier trained in different training contexts (relax, stress, combined) and tested on data from relax and stress context**. The differences between the testing performance for stress and relax context show an interaction between training and test factor: the difficulty of the classifier to generalize to another context. The higher performance for the combined training set relative to the training on data from a single context indicates a gain of the classifier in invariance and hence a protection against over-fitting.

**Figure 8:**
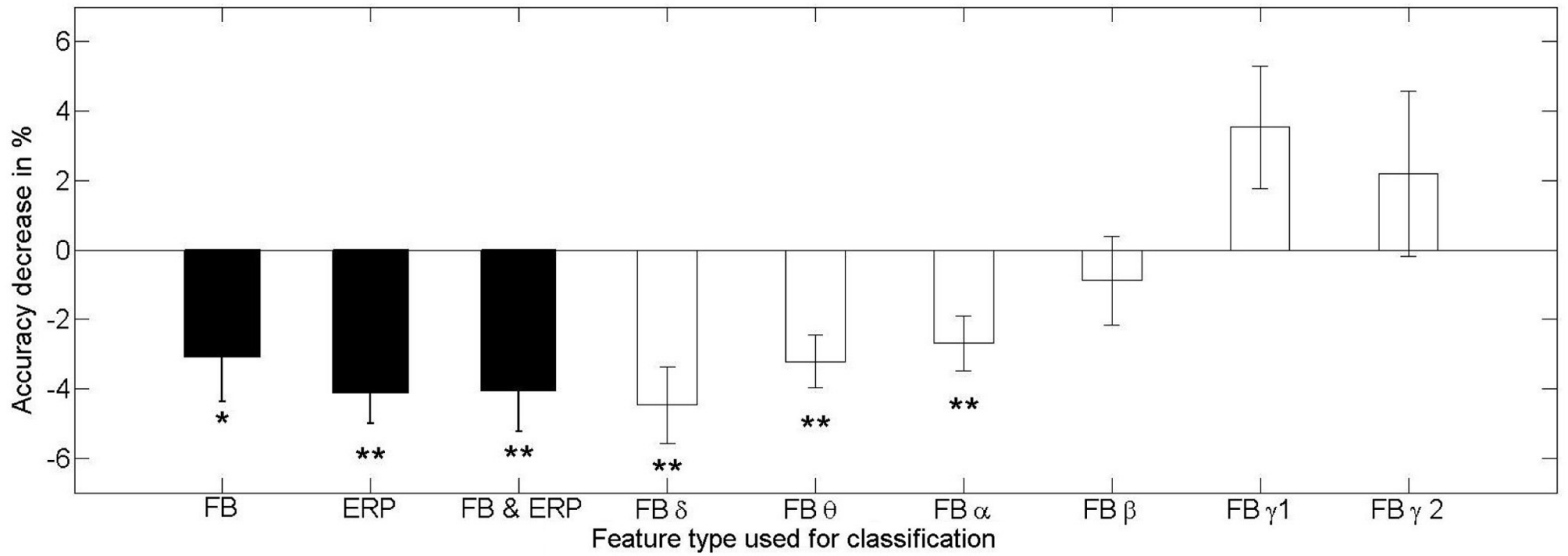
**Mean and standard error of the mean loss of the classification performance of a classifier trained and tested in different training contexts for the feature types from frequency-domain (FB), time-domain (ERP), both (FB and ERP), and for the individual frequency bands (FB α − γ2)**. The stars indicate the significance testing with the respective methods mentioned in the text (^*^p < 0.05, ^**^p < 0.01). Only the low frequency bands show significant effects of performance detoriation in the phase of changing affective context, while the high frequency bands seem relatively stable against changes in context.

In this section we tested the generalization of the classifier to a different affective context (question Q3). To evaluate the effects of testing in dependence of training context, we conducted a 2 (training context: relax, stress) × 2 (testing context: same-as-training, different-from-training) repeated-measures ANOVA for each feature type. Figures 7, 8 depict the average classifier performance when tested within and across affective context and the average loss of performance for the three used feature varieties (and the loss for the specific frequency bands), respectively.

The main effect found for the testing context when using frequency-domain features alone [*F*_(1, 20)_ = 5.610, *p* = 0.028, *η^2^_p_* = 0.219] shows that the transfer from one context to another is problematic and results in a decrease of classifier performance (mean = 69.4 ± 9.7%) compared to testing on the same context as for the training (mean = 72.4 ± 9.4%). An exploratory analysis of the effect of context change on classifiers using only specific frequency bands revealed a significant contribution of the low frequency bands to the performance decline, while the less relevant high frequency bands were not or only minimally contributing (see Figure 8).

For time-domain features alone, the decrease of classifier performance for across context is as well significant, though stronger [*F*_(1, 20)_ = 21.002, *p* < 0.001, *η^2^_p_* = 0.512], with a lower across-context classification performance (mean = 69.1 ± 5.5%) compared to within-context classification performance (mean = 73.3 ± 5.1%).

For frequency- and time-domain features combined, the decrease of classifier performance across-context (mean = 73.2 ± 8.8%) compared within-context (mean = 77.3 ± 7.9%) is as well marked [*F*_(1, 20)_ = 12.104, *p* = 0.002, *η^2^_p_* = 0.377].

To rule out that the differences between within-context and across-context training were caused by the time passing between affective contexts, we divided each context into two parts (1st half, 2nd half) and trained and tested the classifiers in the same manner as done for the within (e.g., training and test on 1st half) and across affective context (e.g., training on 1st half and test on 2nd half) tests. With the data averaged over affective contexts, we conducted a 2 (training context: 1st half, 2nd half) × 2 (testing context: same-as-training, different-from-training) repeated-measures ANOVA for each feature type. We did not find the pattern of performance loss that we observed for within vs. across affective context testing. Surprisingly, the only effect we found was a increase of performance for across vs. within context (half) testing for the frequency-domain only feature variety [*F*_(1, 20)_ = 5.142, *p* < 0.04, *η^2^_p_* = 0.204] from 61.1% to 63.7%.

Summarizing, all feature varieties have been found susceptible to changes in affective context. For the frequency-domain features, only classifiers using the low frequency bands of delta, theta and alpha are significantly declining in performance when tested in an affective context different from the training context (see Figure 8). However, as we showed, these frequency bands are the most informative regarding the workload level. An additional test of the within vs. across effects between the 1st and 2nd half of the affective contexts on classifier performance showed that the time effect alone does not lead to a consistent decrease of performance.

#### Across-context calibration

To evaluate the use of a combined training context to increase the capability of the classifier to generalize over affective contexts (question Q4), we conducted a 2 (training context: average single, combined) × 2 (testing context: stress, relax) repeated-measures ANOVA for each feature type. The specific effects of across-context calibration in comparison to single context (stress and relax) calibration are depicted in Figure 7.

The main effect of the training context for frequency-domain features alone [*F*_(1, 20)_ = 6.816, *p* = 0.017, *η^2^_p_* = 0.254] indicates a higher performance for training with combined (mean = 72.4 ± 9.5%) vs. with single affective context (mean = 70.9 ± 9.3%). There is no significant difference between testing on the (optimal) same context vs. combined testing.

For time-domain features the increase of classifier performance between single (mean = 71.2 ± 5.2%) and combined context (mean = 72.1 ± 4.9%) training is as well significant [*F*_(1, 20)_ = 6.703, *p* = 0.017, *η^2^_p_* = 0.251]. Despite the observed increase due to training with combined data from both contexts, there is still a significant decrease of performance of about 1.2% relative to training and testing on the same context [*t*_(20)_ = − 3.526, *p* < 0.01].

For frequency- and time-domain features combined, we observed an increase of classifier performance between single (mean = 76.7 ± 7.6%) and combined context training (mean = 75.2 ± 8.1%) with [*F*_(1, 20)_ = 6.306, *p* = 0.021, *η^2^_p_* = 0.240]. There is no difference between testing on the (optimal) same context vs. combined testing.

Summarizing, for those classifiers trained with frequency-domain and combined frequency- and time-domain features, training on combined contexts leads to an increase of performance comparable with (optimal) same context training and testing. For classifiers trained with time-domain features only, we observe a significant increase of classification performance when training on combined context, but there is still a loss of performance compared to the (optimal) same context training and testing. Since the number of trials for both conditions are kept equal, this is evidence for a gain in resilience of the workload classifier against contextual changes, especially for classifiers based on frequency-domain features.

## Discussion

If we want to create passive brain-computer interfaces that work in the wild, we need to take the variability of such environments into account. To test how well a workload classifier would be able to cope with variability due to changes in affective context, we trained it on the data from a subject performing a task under the evaluative pressure of an impending interview, the same subject in a non-stressful setting, and from both contexts.

We validated the experimental protocol using subjective and objective indicators of the psychophysiological activation expected due to stress/relaxation induction and different workload levels. Though we did not see a significant difference in the perceived arousal measure (SAM), higher values for the STAI and increased sympathetic nervous system activity (as indicated by significant differences for GSR and a trend for HR) support a successful induction of anxiety in the stressful compared to the non-stressful condition. Higher perceived arousal and mental demand, higher sympathetic nervous system activity (as indexed by HR) as well as lower behavioral performance for high compared to low workload levels support the efficacy of the workload induction paradigm.

We showed that workload can be classified on the basis of 2 s of neurophysiological signals with an accuracy of 76.1%. This is comparable to previously reported results for such short intervals of data (Grimes et al., 2008; Brouwer et al., 2012). It was shown that the accuracy can be increased using decision-level fusion over the results of several trials (Brouwer et al., 2012) or simply by using longer signal epochs (Grimes et al., 2008), however, with the tradeoff of a less fine-grained, more discrete, and lagging measure of workload. We observed a similar increase of classifier performance to between 94.4% and 96% using a majority vote based on the classifier outcome of the relevant 45 trials of a given block.

While the source of information measured via EEG, neuronal or myographical, might seem of no immediate significance for an application on able-bodied users, it seems relevant to us to ensure that we indeed measure the neural activity implied by pBCI. In this regard, it is noteworthy that the distribution of relevant frequencies vary between subjects. While in general the majority of features (65%) is selected from low frequency bands (delta, theta, alpha), some subjects have a strong contribution of high frequencies (beta, gamma, gamma2) up to 50%. Since these higher frequency bands are notorious for their response to muscle activity in addition to neuronal information (Goncharova et al., 2003), we tested if the workload classification would suffer considerably when excluding them from the feature pool. The average performance did indeed decrease slightly to 74.2%. However, the highly significant above-chance performance over all subjects indicates an only marginal role of muscular activity in workload estimation^1^. This is in line with other studies that suggest a relevance of low frequency bands for workload (Jensen et al., 2002; Jensen and Tesche, 2002) and its estimation (Zarjam et al., 2013). Consequently, we showed that the trained classifier uses the neural correlates of workload to discern two workload levels with a performance equaling that reported in similar studies.

Regarding the classifier generalization to different affective contexts, we show that a classifier created in a non-stressful context can generalize to a stressful context and vice versa. However, the training context has a significant influence on the classification performance, with decreasing performance for cross-context classification (i.e., from 72.4% to 69.4% for frequency-domain features, from 73.3% to 69.1% for time-domain features, and from 77.3% to 73.2% for features from both domains). Interestingly, we found that a training which takes several relevant contexts into account enables the generalization of the classifier to a certain degree. Classifiers based on frequency-domain and on combined frequency- and time-domain features perform comparably well after training with data from both affective context (72.4% and 76.7%, respectively) as after being trained and tested within a specific context. Classifiers based on time-domain features profit as well from a training with data from both affective contexts (72.1%), but still show a declined performance relative to optimal, within-context training and testing.

The current study is limited in its generality by the use of a stress induction paradigm which manipulates affective context only once. We chose the TSST because it is a recognized standard of social stress induction and a powerful elicitor that allows to keep stimuli and task comparable during the workload session of stressful and non-stressful condition. However, since we have only two stress conditions and not several interleaved stress conditions, the stress manipulation is synonymous with a change in time, though with a counter-balanced order. Both affective contexts are separated by at least 10 min and we can not exclude that signal changes with time played a role for classifier performance. The analysis of effects of time within the affective contexts, however, did not reveal general performance decreases due to time passing and thus adds to the evidence of context-related performance loss. Similarly, the spread of training blocks over a larger time in combined compared to single testing contexts limits comparability of both performance measures. To ensure that our results hold for stress in specific, interleaved stress induction methods can be used, though a viable experiment length, reliability of stress induction, and comparability of stimuli and task need to be guaranteed.

Another limitation of the paradigm can result from a potential interaction of (psychosocial) stress and workload. For example, impaired cognitive processes or increased engagement in the face of evaluative pressure, could lead to differences in participant performance between affective contexts (Eysenck and Derakshan, 2011). Despite the lack of such interaction effects in our analysis, the possibility of participant's performance-related differences being reflected in brain activity is a general issue that needs to be considered, since such changes in brain activity would be only indirectly related to stress. Therefore, future research needs to identify the processes that are responsible for the signal variability in the face of psychosocial stress. On a related note, other stressors could be manipulated to identify the source of the performance decrease, for example in terms of impaired cognitive processes.

The result of our study suggests that classification performance for passive BCIs can be increased using not only a larger quantity of training data, but by introducing qualitative variations. Here, we varied the stress level of our participants during the task performance. This manipulation is comparable to the variation of the affective context of a task in real-world scenarios, for example task performance under pressure vs. normal task performance. Consequently, to create more reliable BCIs for workload detection, robust against alterations in contextual conditions, such as affective factors (emotions, moods), the training data should include data collected under the relevant contextual conditions.

Zander and Jatzev (2012) found that certain metrics might enable the identification of phases of changed contexts and therefore identify phases were additional calibration might be necessary. One could then use transfer learning (Pan and Yang, 2010) or other re-calibration strategies to enable an adaptation of the transfer algorithm to the new context. However, the suggested metric specifically enables the detection of LOC, which is useful for the detection of perceived LOC and subsequent reliability decrease of active BCIs when environmental and internal factors of the user change. Passive BCIs are not directly related to a feeling of control since they do not enable nor aim at the intentional control of machines. Therefore, for passive BCI one needs other indicators of reliability.

Currently, several groups are investigating the cognitive, affective, and demographic factors that influence active BCI performance (see Lotte et al., 2013). We argue that a similar research program would allow to build more robust passive BCIs by (1) taking into account changes in relevant contextual factors (e.g., stress), (2) by exploring indicators of such changes or the subsequent loss of reliability, and (3) by the exploration of strategies to update the classifier in face of the loss of reliability due to contextual changes.

## Conclusion

The current work has relevance for the development of passive brain-computer interfaces that are able to specifically classify one psychophysiological construct (e.g., workload), while being invariant to others (e.g., stress). We devised and validated a protocol to test the effect of stress on pBCI approaches. We showed that a classifier has trouble transfering from stressful training data to non-stressful test data and vice versa, indicating an influence of affective task context on the performance of a workload classifier. Moreover, we found that the classification profits from the training on a mix of the varied affective task contexts. Such classifiers perform comparably well to those trained and tested on the same affective context. More generally spoken, the results suggest that the classification performance is not only dependent on quantitative factors, such as the numbers of channels, amount of training data, or length of trials, but also on qualitative factors, such as the affective context. This underlines the need for studies that identify such contextual factors and that elucidate ways to deal with detrimental effects related to their influence. Future research and development of workload classification systems using physiological sensors needs to take the contextual factors into account to increase the generality and ecological validity of the system.

### Conflict of interest statement

The authors declare that the research was conducted in the absence of any commercial or financial relationships that could be construed as a potential conflict of interest.

## Footnotes

^1^The validation of the administered stress-induction protocol was presented at the PhyCS 2014 conference (see Jeunet et al., 2014 for more details).

^2^For three subjects, the averaging only contained data from the stress context due to missing blocks in the 2nd half of the relax context.

## References

[B1] Ang K. K. Chin Z. Y. Wang C. Guan C. Zhang H. (2012). Filter bank common spatial pattern algorithm on BCI competition IV datasets 2a and 2b. Front. Neurosci. 6:39 10.3389/fnins.2012.00039 22479236 PMC3314883

[B2] Blankertz B. Lemm S. Treder M. Haufe S. Müuller K.-R. (2010). Single-trial analysis and classification of ERP components—a tutorial. Neuroimage 56, 814–825 10.1016/j.neuroimage.2010.06.048 20600976

[B3] Blankertz B. Tomioka R. Lemm S. Kawanabe M. Müller K. R. (2008). Optimizing spatial filters for robust EEG single-trial analysis. IEEE Signal Process. Mag. 25, 41–56 10.1109/MSP.2008.4408441

[B4] Boucsein W. (ed.). (1992). Electrodermal Indices of Emotion and Stress, chapter 3, in Electrodermal Activity (New York, NY: Springer), 369–391

[B5] Bradley M. M. Lang P. J. (1994). Measuring emotion: the self-assessment Manikin and the semantic differential. J. Behav. Ther. Exp. Psychiatry 25, 49–59 10.1016/0005-7916(94)90063-9 7962581

[B6] Brouwer A. M. Hogervorst M. A. van Erp J. B. F. Heffelaar T. Zimmerman P. H. Oostenveld R. (2012). Estimating workload using EEG spectral power and ERPs in the n-back task. J. Neural Eng. 9:045008 10.1088/1741-2560/9/4/045008 22832068

[B7] Buchanan T. W. Etze J. A. Adolphs R. Tranel D. (2006). The influence of autonomic arousal and semantic relatedness on memory for emotional words. Int. J. Psychophysiol. 61, 23–26 10.1016/j.ijpsycho.2005.10.022 16427713

[B8] Crost N. W. Pauls C. A. Wacker J. (2008). Defensiveness and anxiety predict frontal EEG asymmetry only in specific situational contexts. Biol. Psychol. 78, 43–52 10.1016/j.biopsycho.2007.12.008 18295958

[B9] Dickerson S. S. Kemeny M. E. (2004). Acute stressors and cortisol responses: a theoretical integration and synthesis of laboratory research. Psychol. Bull. 130, 355–391 10.1037/0033-2909.130.3.355 15122924

[B10] Duda R. O. Hart P. E. Stork D. G. (2001). Pattern Recognition, 2nd Edn. New York, NY: Wiley-Interscience

[B11] Erp J. B. F. Veltman H. J. Grootjen M. (2010). Brain-Based indices for user system symbiosis, chapter 12 in Brain-Computer Interfaces: Applying Our Minds to Human-Computer Interaction, eds Tan D. S. Nijholt A. (London: Springer), 201–219

[B12] Eysenck M. W. Derakshan N. (2011). New perspectives in attentional control theory. Pers. Individ. Differ. 50, 955–960 10.1016/j.paid.2010.08.01922364371

[B13] Fairclough S. H. Roberts J. S. (2011). Effects of performance feedback on cardiovascular reactivity and frontal EEG asymmetry. Int. J. Psychophysiol. 81, 291–298 10.1016/j.ijpsycho.2011.07.012 21803081

[B14] Gevins A. Smith M. Leong H. McEvoy L. Whitfield S. Du R. (1998). Monitoring working memory load during computer-based tasks with EEG pattern recognition methods. Hum. Factors 40, 79–91 10.1518/001872098779480578 9579105

[B15] Goncharova I. McFarland D. Vaughan T. Wolpaw J. (2003). EMG contamination of EEG: spectral and topographical characteristics. Clin. Neurophysiol. 114, 1580–1593 10.1016/S1388-2457(03)00093-2 12948787

[B16] Grimes D. Tan D. S. Hudson S. E. Shenoy P. Rao R. P. (2008). Feasibility and pragmatics of classifying working memory load with an electroencephalograph, in Proceedings of CHI 2008 (New York, NY: ACM Press), 835–844

[B17] Hart S. G. Staveland L. (1988). Development of NASA-TLX (Task Load Index): results of empirical and theoretical research, in Human Mental Workload eds Hancock P. A. Meshkati N. (Amsterdam: Elsevier), 139–183

[B18] Hellhammer J. Schubert M. (2012). The physiological response to trier social stress test relates to subjective measures of stress during but not before or after the test. Psychoneuroendocrinology 37, 119–124 10.1016/j.psyneuen.2011.05.012 21689890

[B19] Hewig J. Schlotz W. Gerhard F. Breitenstein C. Luerken A. Naumann E. (2008). Associations of the cortisol awakening response (CAR) with cortical activation asymmetry during the course of an exam stress period. Psychoneuroendocrinology 33, 83–89 10.1016/j.psyneuen.2007.10.004 18022766

[B20] Hoffmann U. Vesin J. Ebrahimi T. (2006). Spatial filters for the classification of event-related potentials, in European Symposium on Artificial Neural Networks (ESANN 2006) (Bruges).

[B21] Jensen O. Gelfand J. Kounios J. Lisman J. E. (2002). Oscillations in the alpha band (9-12 Hz) increase with memory load during retention in a short-term memory task. Cereb. Cortex 12, 877–882 10.1093/cercor/12.8.877 12122036

[B22] Jensen O. Tesche C. D. (2002). Frontal theta activity in humans increases with memory load in a working memory task. Eur. J. Neurosci. 15, 1395–1399 10.1046/j.1460-9568.2002.01975.x 11994134

[B23] Jeunet C. Lotte F. Mühl C. (2014). Design and validation of a mental and social stress induction protocol towards load-invariant physiology-based stress detection, in International Conference on Physiological Computing Systems (Lisbon).

[B24] Julian L. J. (2011). Measures of anxiety: state-trait anxiety inventory (STAI), beck anxiety inventory (BAI), and hospital anxiety and depression scale-anxiety (HADS-A). Arthritis Care Res. 63, S467–S472 10.1002/acr.20561 22588767 PMC3879951

[B25] Kirchner W. K. (1958). Age differences in short-term retention of rapidly changing information. J. Exp. Psychol. 55, 352–358 10.1037/h0043688 13539317

[B26] Kirschbaum C. Pirke K.-M. Hellhammer D. H. (1993). The “Trier Social Stress Test”: a tool for investigating psychobiological stress responses in a laboratory setting. Neuropsychobiology 28, 76–81 10.1159/000119004 8255414

[B27] Krout R. E. (2007). Music listening to facilitate relaxation and promote wellness: integrated aspects of our neurophysiological response to music. Arts Psychother. 34, 134–141 10.1016/j.aip.2006.11.001

[B28] Krusienski D. J. Grosse-Wentrup M. Galán F. Coyle D. Miller K. J. Forney E. (2011). Critical issues in state-of-the-art brain–computer interface signal processing. J. Neural Eng. 8:25002 10.1088/1741-2560/8/2/025002 21436519 PMC3412170

[B29] Lewis R. S. Weekes N. Y. Wang T. H. (2007). The effect of a naturalistic stressor on frontal EEG asymmetry, stress, and health. Biol. Psychol. 75, 224–239 10.1016/j.biopsycho.2007.03.004 17512106

[B30] Loggia M. L. Juneau M. Bushnell M. C. (2011). Autonomic responses to heat pain: heart rate, skin conductance, and their relation to verbal ratings and stimulus intensity. Pain 152, 592–598 10.1016/j.pain.2010.11.032 21215519

[B31] Lotte F. Congedo M. Lecuyer A. Lamarche F. Arnaldi B. (2007). A Review of classification algorithms for EEG-based brain-computer interfaces. J. Neural Eng. 4, R1–R13 10.1088/1741-2560/4/2/R01 17409472

[B32] Lotte F. Guan C. (2010). Learning from other subjects helps reducing brain-computer interface calibration time, in International Conference on Audio, Speech and Signal Processing (ICASSP'2010) (Dallas), 614–617 10.1109/ICASSP.2010.5495183

[B33] Lotte F. Guan C. Ang K. (2009). Comparison of designs towards a subject-independent brain-computer interface based on motor imagery, in International Conference of the IEEE Engineering in Medicine and Biology Society (EMBC) 2009 (Minneapolis), 4543–4546 10.1109/IEMBS.2009.5334126 19964647

[B34] Lotte F. Larrue F. Mühl C. (2013). Flaws in current human training protocols for spontaneous brain-computer interfaces: lessons learned from instructional design. Front. Hum. Neurosci. 7:568 10.3389/fnhum.2013.00568 24062669 PMC3775130

[B35] Mathan S. Feyereisen T. Whitlow S. (2007). WorkSense: exploring the feasibility of human factors assessment using electrophysiological sensors, in Proceedings of ICACS (Santa Monica).

[B36] Müller-Putz G. R. Scherer R. Brunner C. Leeb R. Pfurtscheller G. (2008). Better than random? A closer look on BCI results. Int. J. Bioelectromagn. 10, 52–55

[B37] Pan S. J. Yang Q. (2010). A survey on transfer learning. IEEE Trans. Knowl. Data Eng. 22, 1345–1359 10.1109/TKDE.2009.191

[B38] Peck E. M. M. Yuksel B. F. Ottley A. Jacob R. J. Chang R. (2013). Using fNIRS brain sensing to evaluate information visualization interfaces, in Proceedings of CHI 2013 (New York, NY: ACM Press), 473–482

[B39] Peng H. Long F. Ding C. (2005). Feature selection based on mutual information: criteria of max-dependency, max-relevance, and min-redundancy. IEEE Trans. Pattern Anal. Mach. Intell. 27, 1226–1238 10.1109/TPAMI.2005.159 16119262

[B40] Reinhardt T. Schmahl C. Wüst S. Bohus M. (2012). Salivary cortisol, heart rate, electrodermal activity and subjective stress responses to the Mannheim Multicomponent Stress Test (MMST). Psychiatry Res. 198, 106–111 10.1016/j.psychres.2011.12.009 22397919

[B41] Renard Y. Lotte F. Gibert G. Congedo M. Maby E. Delannoy V. (2010). OpenViBE: an open-source software platform to design, test, and use brain-computer interfaces in real and virtual environments. Presence Teleop. Virt. 19, 35–53 10.1162/pres.19.1.35

[B42] Reuderink B. Mühl C. Poel M. (2013). Valence, arousal and dominance in the EEG during game play. Int. J. Auton. Adapt. Commun. Syst. 6:45 10.1504/IJAACS.2013.050691

[B43] Reuderink B. Poel M. Nijholt A. (2011). The impact of loss of control on movement BCIs. IEEE Trans. Neural Syst. Rehab. Eng. 19, 628–637 10.1109/TNSRE.2011.2166562 21984517

[B44] Roy R. Bonnet S. Charbonnier S. Campagne A. (2012). Time-on-task effect on workload level discriminability through electroencephalography, in Proceedings of BIOMAG 2012 (Paris).

[B45] Schlögl A. Keinrath C. Zimmermann D. Scherer R. Leeb R. Pfurtscheller G. (2007). A fully automated correction method of eog artifacts in eeg recordings. Clin. Neurophysiol. 118, 98–104 10.1016/j.clinph.2006.09.003 17088100

[B46] Selye H. (1936). A syndrome produced by diverse nocuous agents. Nature 138:32 10.1038/138032a0 9722327

[B47] Sinha R. Talih M. Malison R. Cooney N. Anderson G.-M. Kreek M.-J. (2003). Hypothalamic-pituitary-adrenal axis and sympatho-adreno-medullary responses during stress-induced and drug cue-induced cocaine craving states. Psychopharmacology 170, 62–72 10.1007/s00213-003-1525-8 12845411

[B48] Solovey E. Schermerhorn P. Scheutz M. Sassaroli A. Fantini S. Jacob R. (2012). Brainput: enhancing interactive systems with streaming fnirs brain input, in Proceedings of CHI 2012 (Austin, TX), 2193–2202

[B49] Spielberger C. D. Gorsuch R. L. Lushene R. E. (1970). Manual for the State-Trait Anxiety Inventory. Palo Alto, CA: Consulting Psychologists Press

[B50] Tangermann M. Müller K. R. Aertsen A. Birbaumer N. Braun C. Brunner C. (2012). Review of the BCI competition IV. Front. Neurosci. 6:55 10.3389/fnins.2012.00055 22811657 PMC3396284

[B51] Taniguchi K. Nishikawa A. Sugino T. Aoyagi S. Sekimoto M. Takiguchi S. (2009). Method for Objectively Evaluating Psychological Stress Resulting When Humans Interact with Robots. China: InTech

[B52] Tops M. van Peer J. M. Wester A. E. Wijers A. A. Korf J. (2006). State-dependent regulation of cortical activity by cortisol: an EEG study. Neurosci. Lett. 404, 34–39 10.1016/j.neulet.2006.05.038 16822613

[B53] van Erp J. Lotte F. Tangermann M. (2012). Brain-computer interfaces: beyond medical applications. IEEE Comput. 45, 26–34 10.1109/MC.2012.107

[B54] Verwey W. B. Veltman H. A. (1984). Detecting short periods of elevated workload : a comparison of nine workload assessment techniques. J. Exp. Psychol. Appl. 2, 270–285 10.1037/1076-898X.2.3.270

[B55] Walter C. Rosenstiel S. S., W. Gerjets P. Bogdan M. (2013). Using cross-task classification for classifying workload levels in complex learning tasks, in Proceedings of ACII 2013 (Geneva), 876–881

[B56] Zander T. Jatzev S. (2012). Context-aware brain–computer interfaces: exploring the information space of user, technical system and environment. J. Neural Eng. 9:016003 10.1088/1741-2560/9/1/016003 22156069

[B57] Zander T. O. Kothe C. (2011). Towards passive brain-computer interfaces: applying brain-computer interface technology to human-machine systems in general. J. Neural Eng. 8:025005 10.1088/1741-2560/8/2/025005 21436512

[B58] Zarjam P. Epps J. Chen F. Lovell N. H. (2013). Estimating cognitive workload using wavelet entropy-based features during an arithmetic task. Comput. Biol. Med. 43, 2186–2195 10.1016/j.compbiomed.2013.08.021 24290935

[B59] Zijlstra F. R. H. (1993). “Efficiency in Work Behaviour: A Design Approach for Modern Tools,” Ph.D. thesis, Delft University of Technology.

